# Walnut peptide alleviates obesity, inflammation and dyslipidemia in mice fed a high-fat diet by modulating the intestinal flora and metabolites

**DOI:** 10.3389/fimmu.2023.1305656

**Published:** 2023-12-14

**Authors:** Lei Li, Si Wang, Tong Zhang, Bijun Lv, Yanling Jin, Yue Wang, Xiaojiao Chen, Ning Li, Niping Han, Yueying Wu, Jiali Yuan

**Affiliations:** ^1^ College of Basic Medical Sciences, Yunnan University of Chinese Medicine, Kunming, Yunnan, China; ^2^ Yunnan Provincial Key Laboratory of Integrated Traditional Chinese and Western Medicine for Chronic Disease in Prevention and Treatment, Yunnan University of Chinese Medicine, Kunming, Yunnan, China; ^3^ First Clinical School of Medicine, Yunnan University of Chinese Medicine, Kunming, Yunnan, China

**Keywords:** medicinal homologous food, obesity, walnut peptide, intestinal flora, inflammation, lipid metabolism

## Abstract

**Introduction:**

Obesity is a chronic disease in which the body stores excess energy in the form of fat, and intestinal bacterial metabolism and inflammatory host phenotypes influence the development of obesity. Walnut peptide (WP) is a small molecule biopeptide, and the mechanism of action of WP against metabolic disorders has not been fully elucidated. In this study, we explored the potential intervention mechanism of WP on high-fat diet (HFD)-induced obesity through bioinformatics combined with animal experiments.

**Methods:**

PPI networks of Amino acids and their metabolites in WP (AMWP) and “obesity” and “inflammation” diseases were searched and constructed by using the database, and their core targets were enriched and analyzed. Subsequently, Cytoscape software was used to construct the network diagram of the AMWP-core target-KEGG pathway and analyze the topological parameters. MOE2019.0102 was used to verify the molecular docking of core AMWP and core target. Subsequently, an obese Mice model induced by an HFD was established, and the effects of WP on obesity were verified by observing weight changes, glucose, and lipid metabolism levels, liver pathological changes, the size of adipocytes in groin adipose tissue, inflammatory infiltration of colon tissue, and intestinal microorganisms and their metabolites.

**Results:**

The network pharmacology and molecular docking showed that glutathione oxide may be the main active component of AMWP, and its main targets may be EGFR, NOS3, MMP2, PLG, PTGS2, AR. Animal experiments showed that WP could reduce weight gain and improve glucose-lipid metabolism in HFD-induced obesity model mice, attenuate hepatic lesions reduce the size of adipocytes in inguinal adipose tissue, and reduce the inflammatory infiltration in colonic tissue. In addition, the abundance and diversity of intestinal flora were remodeled, reducing the phylum Firmicutes/Bacteroidetes (F/B) ratio, while the intestinal mucosal barrier was repaired, altering the content of short-chain fatty acids (SCFAs), and alleviating intestinal inflammation in HFD-fed mice. These results suggest that WP intervenes in HFD-induced obesity and dyslipidemia by repairing the intestinal microenvironment, regulating flora metabolism and anti-inflammation.

**Discussion:**

Our findings suggest that WP intervenes in HFD-induced obesity and dyslipidemia by repairing the intestinal microenvironment, regulating flora metabolism, and exerting anti-inflammatory effects. Thus, WP may be a potential therapeutic strategy for preventing and treating metabolic diseases, and for alleviating the intestinal flora disorders induced by these diseases. This provides valuable insights for the development of WP therapies.

## Graphical Abstract

WP intervenes in HFD-induced obesity and dyslipidemia by repairing the intestinal microenvironment, regulating flora metabolism and anti-inflammation.
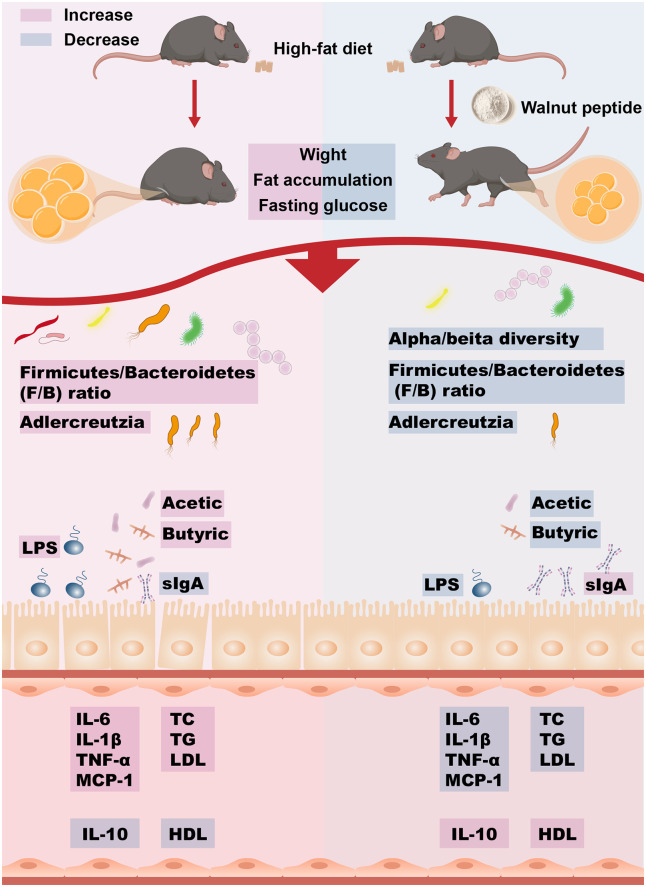

## 1 Introduction

Energy imbalance or metabolic changes in the body are the cause of excessive weight gain and accumulation of body adipose, leading to obesity, which is defined by the WHO as a BMI ≥30.0 kg/m² (1). Obesity is an important factor in the increasing mortality associated with non-communicable diseases (NCDs) by inducing metabolic disorders in the body, which ultimately lead to the development of multi-system diseases (2). From 2002 to 2018, the prevalence of obesity in the Chinese population rose from 7.1 percent to 16.4 percent, and the resulting healthcare burden is estimated to be as high as RMB 418 billion, accounting for 21.5 percent of total healthcare expenditure (3). At the same time, due to differences in comorbidity, mortality, and body composition data, the applicable threshold for BMI obesity appears to be lower in the Chinese than in Europe and the United States, at 25.9 kg/m² and 26.6 kg/m² for men and women, respectively (4, 5). Therefore, it is even more important for Chinese people to intervene in advance to avoid the development of obesity.

Chronic low-grade inflammatory state characterizes obesity and intestinal microenvironmental changes interact with low-grade inflammation (6). Firstly, inflammation is an important causative factor for the imbalance of intestinal microenvironmental homeostasis in obese people, and secondly, the dysregulation of intestinal flora and over-colonization of harmful flora can damage the integrity of the intestinal mucosal barrier, resulting in intestinal microecological disorders, and the leakage of microbial lipopolysaccharides (LPS) and other toxins into the bloodstream, resulting in metabolic endotoxemia, which can aggravate the inflammatory state of the obese people (7–9).

Walnut is a kind of medicinal and food tree nut rich in many nutrients, WP is a small molecule bio-peptide extracted from walnut meal after walnut oil extraction, with active functions such as anti-oxidative stress, anti-hypertension, neuroprotection, blood glucose regulation, anti-cancer and anti-hyperuricemia, anti-inflammation, etc. (10, 11). In addition, YANG X-Y et al. found that WP was able to reduce body weight and improve glucose-lipid metabolism in rats on an HFD (12), however, the mechanism of action has not been fully elucidated.

Given the relationship between intestinal microecology, inflammation, and obesity, this study aims to investigate whether WP can mitigate weight gain and glucose-lipid metabolism abnormalities in mice subjected to a HFD induced obesity model. This is achieved by improving intestinal bacterial dysbiosis, repairing damage to the intestinal mucosal barrier, and remodeling the intestinal microenvironment. This, in turn, reduces the body’s inflammation levels. The investigation will observe changes in relevant indices following the administration of different WP dosages in mice on an HFD.

## 2 Materials and methods

### 2.1 UPLC-MS/MS

50 mg (± 2.5 mg) of WP (PB01401, Shaanxi Pioneer Biotech Co., Ltd.) was weighed, and 500 μL of pre-cooled 70% methanol aqueous extract at -20 ℃ was immediately added, vortexed, and centrifuged, and 200 μL of the supernatant after centrifugation was passed through a protein precipitation plate for on-line analysis. The data acquisition instruments mainly included Ultra Performance Liquid Chromatography (UPLC) (ExionLC™ AD) and Tandem Mass Spectrometry (MS/MS) (QTRAP® 6500+). The data detected by mass spectrometry were analyzed qualitatively and quantitatively (n = 3) based on the construction of a Met ware Database (MWDB) database based on the standards and the Multiple Reaction Monitoring (MRM) mode using triple quadrupole mass spectrometry.

### 2.2 Network pharmacological analysis and molecular docking validation

#### 2.2.1 Target prediction

WP active ingredient targets of action and obesity, inflammation-related targets were collected through PubChem (https://pubchem.ncbi.nlm.nih.gov/), SwissTargetPrediction (http://ww-w.swisstargetprediction.ch/), UniProt (https://www.uniprot.org), GeneCards (https://www.genecards.org), OMIM (http://www.omim.org) and other databases were searched for prediction and screening.

#### 2.2.2 Construction of PPI (protein-protein interaction) networks

Venn diagrams were produced online using Venny 2.1.0 (https://bioinfogp.cnb.csic.es/tools/venny/) to obtain the intersection targets of active ingredient action targets with obesity and inflammation, and the String11.5 (https://cn.string-db.org/) database, constructed PPI networks, and the results were imported into Cytoscape 3.7.1 software for visualization, and 50 core targets of WP modulating inflammation to ameliorate obesity were screened by MCC algorithm using CytoHubba plug-in.

#### 2.2.3 GO functional analysis and KEGG pathway enrichment analysis

The 50 core targets were imported into the Metascape (https://metascape.org/gp/index.html) database for GO functional analysis and KEGG pathway enrichment analysis.

#### 2.2.4 AMWP-core target-KEGG pathway network map construction

The AMWP, 50 core targets, and KEGG pathway results in CytoScape 3.7.1 were used to construct a network map, and the network topology parameters were analyzed using the software’s inbuilt tool, Network Analyzer, and screened to play a role in ranking the top 10 core components and the top 6 key target proteins according to the degree value and molecular docking validation.

#### 2.2.5 Molecular docking validation

The PDB files of the key target proteins were downloaded from the RCSB PDB (http://www.rcsb.org/) database, the protein source was “human”, and the 2D structure of the core component was downloaded from the PubChem database. MOE2019.0102 was used to optimize the energy of the small molecules, the protein receptor was dehydrogenated and demineralized, and the binding strength and activity of the key target and the core component were evaluated according to the Docking Score value.

### 2.3 Animals and treatments

Twenty-five 8-week-old male C57BL/6J mice were purchased from China Spefo (Beijing) Biotechnology Co Ltd. (Laboratory Animal Licence No. SYXK[Beijing]K2019-0010). Animal experiments were approved by the Animal Ethics Experimentation Committee by the National Institutes of Health Guide for the Care and Use of Laboratory Animals (Approval No. R-062021084). Throughout the experiments, mice were housed in the SPF-grade Animal Experimentation Centre of Yunnan University of Traditional Chinese Medicine, where animals were kept at a controlled temperature (23°C ± 1°C) under a 12-h light-dark cycle with water and food supplied ad libitum. Twenty-five mice were randomly divided into control, model, and WP low-dose, WP medium-dose, and WP high-dose groups (n = 5 in each group). The control group was fed a control diet (NC, MD12031, Medicience, Professionals for Lab Animal Diets), and the model and WP groups were fed an HFD (HFD, MD12032, Medicience, Professionals for Lab Animal Diets). Mice in the WP group were given WP (220 mg/kg/d, 440 mg/kg/d, 880 mg/kg/d) dissolved in saline by gavage during the feeding period, and the rest of the mice were given saline by gavage at 0.1 ml/g/d for 10 weeks. 8-week-old C57BL/6J male mice are sensitive animal models of diet-induced obesity. It is generally believed that the simple modeling of obesity is the body weight of the experimental group is 20% more than that of the control group. Follow the Replace, Reduce, Refine principle (eliminate serum testing of drugs, use the fewest animals for statistical analysis) to minimize animal damage. Mice were anesthetized with 3% isoflurane (Shenzhen Reward Life Technology Co., Ltd.) at week 10, as recommended in animal ethical guidelines. Blood was quickly collected from the abdominal aorta, intestinal and liver tissue was collected, and the body was disposed of according to recommended guidelines. When the mice had neither a heartbeat nor any nerve reflexes, the mice were considered sacrificed.

### 2.4 Determination of serum TC and TG by COD-PAP method

The total cholesterol assay kit (A111-1-1) was placed at room temperature for 15 min to establish blank, standard, and sample wells. The blank wells were filled with 2.5 μL of pure water, the standard wells were filled with 2.5 μL of standard, and the sample wells were filled with 2.5 μL of serum samples, 250 μL of the working solution was added to each well, and the wells were incubated at 37°C for 10 min. The absorbance of each well was measured at 510 nm using the enzyme marker, and the value was substituted into the formula for the calculation of the TC content. The triglyceride assay kit (A110-1-1) was used to detect TG content as above.

### 2.5 Determination of serum LDL-C and HDL-C in mice by direct method

A low-density lipoprotein cholesterol assay kit (A113-1-1) was placed at room temperature for 15 min to establish blank, standard and the blank, standard, and sample wells were established. The blank wells were filled with 2.5 μL of water, the standard wells were filled with 2.5 μL of standard, the sample wells were filled with 2.5 μL of serum, and each well was filled with 180 μL of LDL-C reagent A. The sample wells were incubated at 37℃ for 5 min, and the absorbance at 546 nm was measured (A1). Each well was filled with 60 μL of LDL-C reagent B, and each well was incubated at 37°C for 5 min, and the absorbance at 546 nm was measured (A2), and the LDL-C content was obtained by the equation. High-density lipoprotein cholesterol assay kit (A113-1-1) was used to determine HDL-C content as above. All test kits are purchased from Nanjing Jiancheng Bioengineering Institute.

### 2.6 Pathological section

Hematoxylin-eosin staining and Oil Red O staining were performed on the liver tissue. For Hematoxylin-eosin staining, liver, adipose tissue, and colon tissues were taken, dehydrated, and embedded in paraffin. The tissue sections were then treated with environmentally friendly dewaxing solutions I and II, followed by anhydrous ethanol I and II. After rinsing with 75% alcohol and clean water, the frozen sections were warmed, fixed, stained with hematoxylin and eosin, and dehydrated with three rounds of anhydrous ethanol and two rounds of xylene. Finally, the sections were sealed with neutral gum and analyzed using a Nikon Eclipse ci microscope with NIS_F_Ver43000_64bit_E software and a NIKON digital sight DS-FI2 imaging system. For Oil Red O staining, liver tissue was fixed, embedded in OCT, and frozen sections were prepared. The sections were then stained with Oil Red O solution, differentiated with isopropyl alcohol, and rinsed with distilled water. Hematoxylin staining and reblueing were performed, followed by sealing the sections with glycerol gelatin sealant. The sections were observed under a microscope and images were collected and analyzed.

H&E staining provides information about the structure, pathological changes, and inflammation of liver cells. By comparing the H&E staining results of obese and normal mice, the impact of obesity on the liver can be assessed. H&E staining of adipose tissue allows for a visual assessment of changes in the structure and size of adipocytes. Oil Red O staining is used to evaluate the accumulation of fat in liver tissue. Through staining, neutral fats in the liver tissue are stained red, allowing for analysis of the presence of fat droplets in the liver tissue of obese mice. Additionally, H&E staining of colon tissue in obesity mouse models provides information about morphological changes and inflammation. This aids in studying the relationship between obesity and colon health.

### 2.7 Immunohistochemistry

Paraffin sections of colon tissue were dewaxed to water for antigenic repair, the antibody information and repair conditions are shown in **Supplementary Material 1**, cooled naturally, and washed three times with PBS (pH 7.4) for 5 min each time. The sections were incubated in 3% hydrogen peroxide solution for 25 min at room temperature, protected from light, and washed again with PBS. The slices were blocked with 3% BSA for 30 min at room temperature. The blocking solution was discarded, a drop of the primary antibody was added to the section, and the section was incubated overnight at 4°C in the flat area of the humid box. The sections were incubated in a wet box at 4°C overnight. After the sections were washed, the corresponding secondary antibody (**Supplementary Material 2** for the secondary antibody’s product number, manufacturer, and dilution ratio) was added dropwise in the circle to cover the tissues, and the sections were incubated at room temperature for 50 min. DAB was used to develop the color, and the time of color development was controlled under the microscope, A positive color was brownish-yellow, and the color development was terminated by rinsing the sections with tap water. The nuclei were stained with hematoxylin, the slices were dehydrated and sealed, and the results were interpreted under a white light microscope.

### 2.8 Determination of sIgA, LPS, and inflammatory factors in serum and colonic tissue

Serum was obtained by centrifugation of mice whole blood at 4°C for 10 min after 2-4 h at room temperature. Approximately 30 mg of colon tissue was removed, rinsed with 9 times the volume of phosphate-buffered saline, homogenized in a tissue grinder, and centrifuged at 4°C for 15 min. Remove the supernatant and place it on ice. Mice secretory immunoglobulin A (sIgA), lipopolysaccharide (LPS), monocyte chemotactic protein-1 (MCP-1), tumor necrosis factor α (TNF-α), interleukin 6 (IL-6), interleukin 10 (IL-10), interleukin 1β (IL-1β) enzyme-linked immunosorbent assay (ELISA) kit (Enzyme Labeling BioTech Co. Ltd.) was allowed to stand at room temperature for 30 min, and the standard wells, blank control wells and sample wells were set according to the instructions. Standards were added to the standard wells, dilutions and samples were added to all sample wells. Except for the blank wells, 100 μL of horseradish peroxidase (HRP)-labeled assay antibody was added to each standard and sample well. Incubate in a sealed incubator at 37°C for 1 hour. The plate was washed 5 times. Add color development solutions A and B to each well and develop the plate for 15 min at 37°C in a sealed incubator protected from light. Add the termination solution and measure the absorbance value at 450 nm using an enzyme marker.

### 2.9 16S rRNA measurement of mice intestinal flora

Through the regulation of body weight and blood lipids, we found notable intervention effects in all three WP dosage groups, with the highest dose group of WP showing the most significant results. Consequently, only the high-dose group of WP was selected for further exploration of the mechanisms related to intestinal microbiota and their metabolic products. Mice feces were collected under aseptic conditions and stored in liquid nitrogen. The sample DNA was extracted and the V3+V4 variable region was amplified by PCR with primers 341F (5’-CCTAYGGGRBGCASCAG-3’) and 806R (5’-GGACTACNNGGGGTATCTAAT-3’). The V3+V4 variable region was amplified by PCR. The PCR products were purified and mixed. The library was constructed using the NEB Next® Ultra DNA Library Prep Kit. The constructed library was detected and quantified by Q-PCR using an Agilent 5400 and then sequenced online using a NovaSeq 6000. After sequencing, the data were analyzed.

### 2.10 GC-MS determination of SCFAs

The mice fecal samples were thawed on ice, and 30 mg of the samples were taken into a 2 mL glass centrifuge tube. Add 900 μL of 0.5% phosphoric acid, resuspend, shake and mix for 2 min, centrifuge at 14000 g for 10 min, take 800 μL of supernatant, add an equal amount of ethyl acetate extraction, shake and mix for 22 min, centrifuge at 14000 g for 10 min, take 600 μL of the upper layer of the organic phase, add 500 μm 4-methyl pentanoic acid as the internal standard as the final concentration, and then mix well and add into the injection vial, enter into the GC-MS detection, the injection volume of 1 μL, and the split ratio of 10. The sample was injected into the GC-MS with an injection volume of 1 μL and a split ratio of 10:1. The samples were separated on an Agilent DB- FFAP capillary column (30 m x 250 μm x 0.25 μm) using a gas chromatographic system. Mass spectrometry analysis was performed using a 5977B MSD mass spectrometer (Agilent). The peak areas and retention times were extracted by MSD ChemStation software. The calibration curves were plotted and the contents of SCFAs in the samples were calculated.

### 2.11 Statistical analysis

All data were expressed as mean ± standard deviation using GraphPad 9.4.1 software. One-way ANOVA (one-way analysis of variance) was used to make multiple group comparisons, Dunnett’s T3 method was used to make group comparisons if the variance was not uniform, and the LSD-t method was used to make group comparisons if the variance was uniform. *P*<0.05 indicated that the difference was statistically significant.

## 3 Results

### 3.1 WP active ingredient analysis

**Figure 1:**
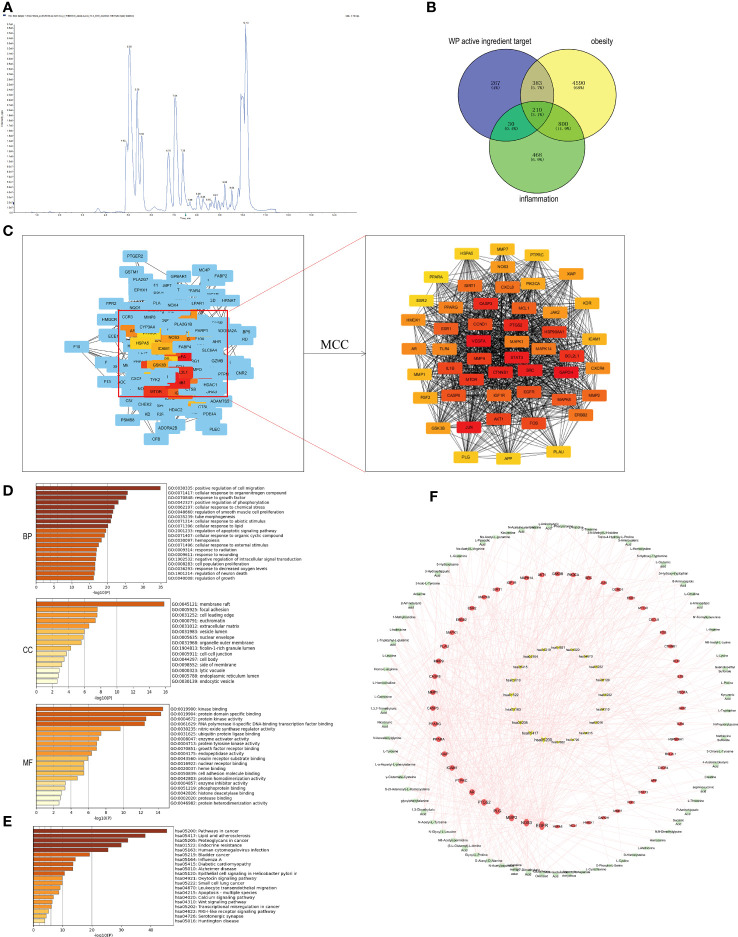
Network pharmacological analysis of WP active components and targets of obesity and inflammatory diseases. **(A)** TIC plot of WP detected by LC-MS/MS (n=3), **(B)** Venn diagram of the intersection of WP active ingredient targets of action with targets of obesity and inflammation, **(C)** CytoHubb screening of the top 50 core targets of WP active ingredient targets of action with targets of obesity and inflammation, **(D)** GO enrichment analysis results, **(E)** KEGG enrichment analysis results, **(F)** AMWP-core target-KEGG pathway network diagram (rectangle is the AMWP corresponding to the 50 core targets, circle is the 50 core targets, inverted triangle is the signaling pathway, the larger the node area, the higher the representation value, the more important the node is in the network).

Based on the LC-MS/MS platform, the targeted quantitative detection of the drug AMWP used for animal experiments, a TIC diagram was obtained, shown in **Figure 1A**. Seventy-seven amino acids and their metabolites including the essential human amino acids L-Tryptophan, L-Leucine, L-Phenylalanine, L-Lysine, L-Valine, L-Isoleucine, L-Methionine, L-Threonine, and L-Histidine, were identified, as shown in **Supplementary Material 3**.

### 3.2 Network pharmacological analysis and molecular docking

#### 3.2.1 Construction of blood entry component targets, obesity disease targets, and PPI networks

890 WP active ingredient action targets, 5983 obesity-related targets, and 1508 inflammation-related targets were obtained from the relevant databases. The intersection of the three targets was taken and the Venn diagram was plotted to obtain 210 common potential targets, as shown in **Figure 1B**. Among them, 50 targets, including GAPDH, VEGFA, JUN, STAT3, and SRC, were the core targets in the PPI network, as shown in **Figure 1C**.

#### 3.2.2 GO, KEGG enrichment analysis

After enrichment of the core targets by the Metascape platform, it was known that the biological process (BP) involved in the improvement of obesity by WP included the cellular response to organic nitrogen compounds, positive regulation of phosphorylation, cellular response to lipids, and regulation of apoptotic signaling pathways, etc., and the related cellular component (CC) included membrane rafts, nuclear membranes, endoplasmic reticulum lumen, intracellular vesicles, etc., and the related molecular function (MF) included protein kinase activity, nitric oxide synthase activity, endoplasmic reticulum lumen, and intracellular vesicles. The relevant CC include membrane rafts, nuclear membrane, endoplasmic reticulum lumen, intracellular vesicles, etc., and the relevant MF includes protein kinase activity, nitric oxide synthase regulatory activity, insulin receptor substrate binding, cell adhesion molecule binding, etc., shown in **Figure 1D**. The results of KEGG enrichment analysis indicated that the signaling pathways involved in the improvement of obesity by WP mainly include lipid and atherosclerosis, endocrine resistance, diabetic cardiomyopathy, etc., shown in **Figure 1E**.

#### 3.2.3 Construction and analysis of the network diagram of the AMWP-core target-KEGG pathway

The network diagram of the AMWP-core target-KEGG pathway was constructed by CytoScape 3.7.1 and is shown in **Figure 1F**. According to the analysis of topological parameters, the top 10 key target proteins were EGFR, NOS3, MMP2, PLG, PTGS2, AR, PTPRC, ICAM1, PPARG, XIAP, PPARA, CASP3, MMP9, CASP8, MMP1, and MAPK1, and the top 10 AMWPs were oxidized glutathione, 3,7-dimethyl uric acid, 5-L-glutamyl-L-alanine, alanyl-alanine, glycyl-L-proline, N-acetyl aspartic acid, methyl tyrosine, N-glycyl-L-leucine, glycyl phenylalanine, and N-acetyl-L-tyrosine.

#### 3.2.4 Molecular docking verification

**Figure 2:**
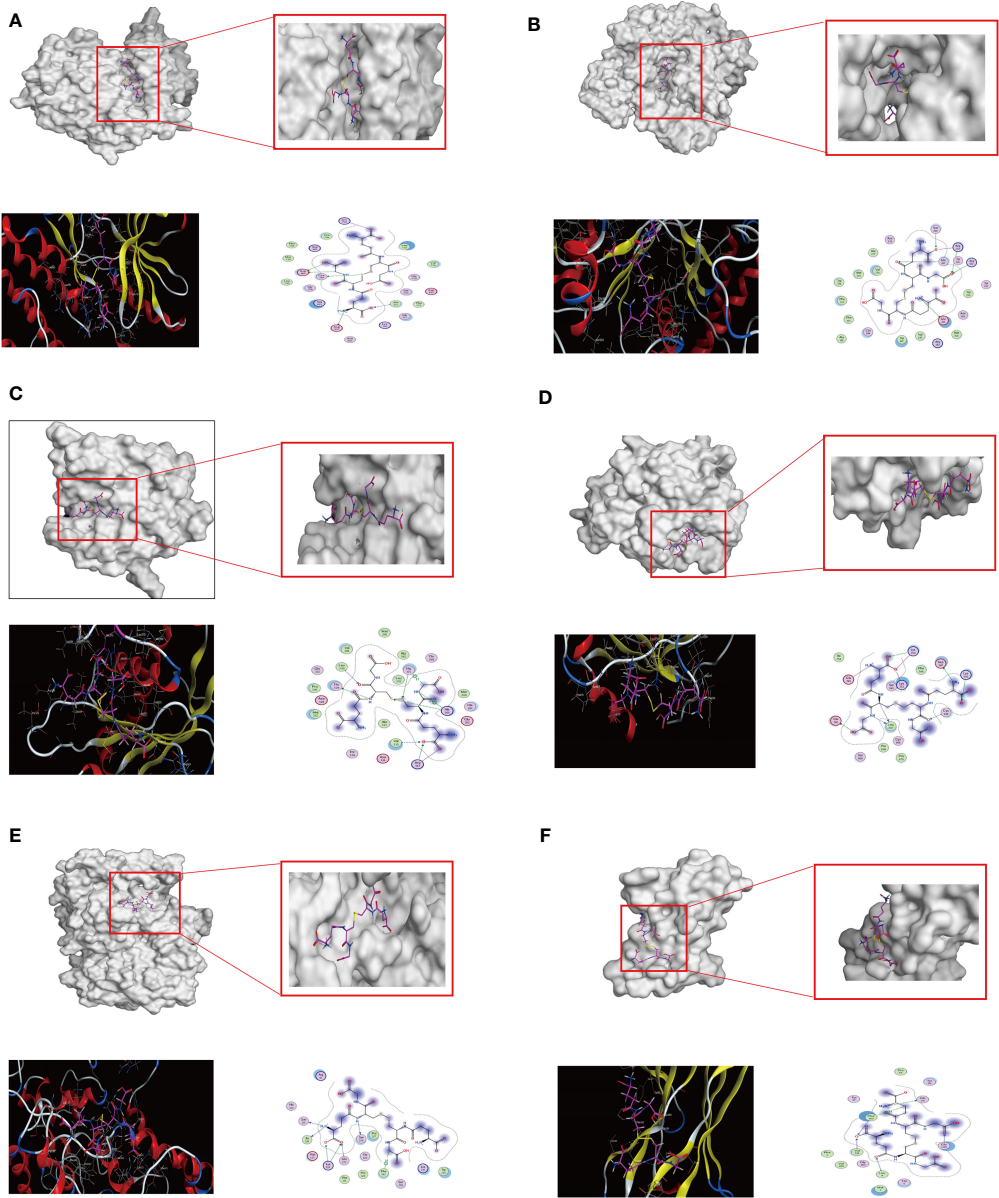
The results of the molecular docking between the key target proteins with the top 6 Degree values and the top 10 AMWP core components. **(A–F)** molecular docking conformation diagrams of oxidized glutathione and EGFR, NOS3, MMP2, PLG, PTGS2, AR. Docking conformational maps.

The molecular docking between the key target proteins with the top 6 Degree values and the top 10 AMWP core components showed that the Docking Score values were all negative, and the results are shown in **Table 1**, which indicated that the AMWP core components had good binding activities with the key target proteins, and that oxidized glutathione had the best degree of binding with the key target proteins, and the Docking Score of all of them was less than -5, which means that the binding of both of them was spontaneous, as shown in **Figures 2A–F**.

**Table 1:** Evaluation table of binding energy between core components and key target proteins.

ingredient	Interface score S value					
	EGFR (7JXQ)	NOS3 (5UO8)	MMP2 (4WKE)	PLG (7THS)	PTGS2 (5F19)	AR (5IR3)
Oxidized glutatdione	-7.8018	-8.3651	-7.9331	-6.9188	-8.1130	-7.8867
3, 7-dimethyluric acid	-5.7490	-4.4714	-5.3898	-5.3961	-4.8150	-4.5627
5-L-glutamyl-L-alanine	-6.5701	-5.5945	-6.2542	-5.3298	-5.6724	-4.9796
N-glycinyl-L-leucine	-5.6053	-5.1765	-5.9476	-4.7692	-5.2937	-4.5997
N-acetyl-L-tyrosine	-5.0309	-5.8435	-6.5033	-5.3781	-5.3621	-4.7172
N-acetylaspartate	-5.5066	-4.8257	-5.3776	-5.0054	-4.9935	-4.6099
Alanyl-alanine	-5.7899	-5.0856	-5.8598	-4.4511	-4.99	-4.3599
Glycyll-L-proline	-5.7421	-4.7969	-5.2111	-4.5061	-4.97	-4.3118
Glycylphenylalanine	-6.7027	5.3553	-6.3287	-5.1495	-5.56	-4.6374
Tyrosine methyl ester	-5.6627	-5.6123	-6.2367	-4.5664	-4.90	-4.3895

### 3.3 WP reduces body weight gain and improves glycolipid metabolism in HFD-fed mice

**Figure 3:**
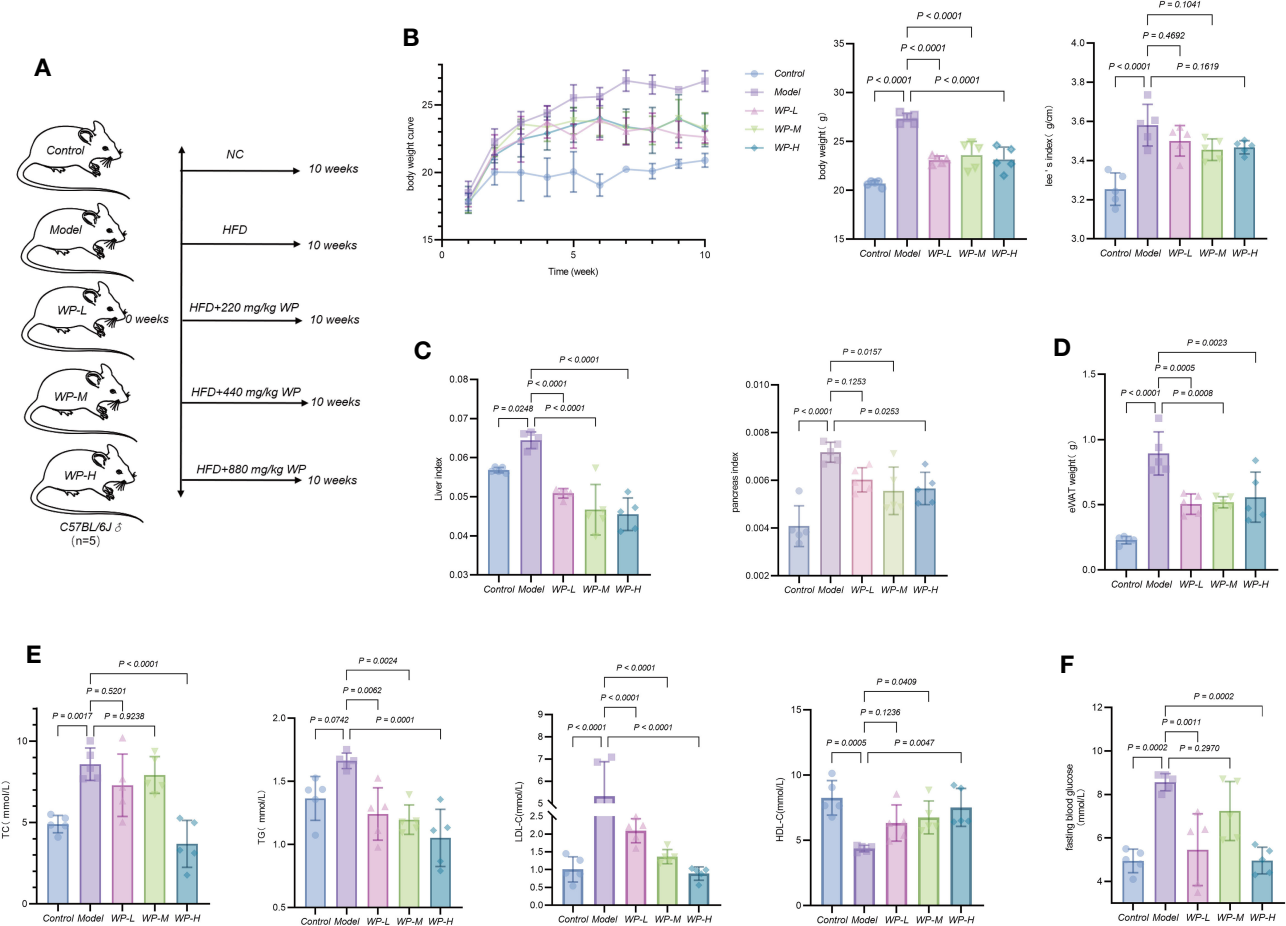
WP reduces body weight gain and improves glycolipid metabolism in HFD-induced obesity model mice. **(A)** Grouping of mice and mode of intervention, **(B)** Weighing method to detect the effect of WP on body weight change and Lee’s index in mice, **(C)** Weighing method to detect the effect of WP on liver coefficient and pancreas coefficient in mice, **(D)** Weighing method to detect the effect of WP on the weight of WAT of epididymal testis in mice, **(E)** COD-PAP method to detect the effect of WP on the serum levels of TC and TG content, Direct method to detect the effect of WP on LDL-C and HDL-C content in mice serum, **(F)** Glucometer to detect the effect of WP on fasting blood glucose level in mice. (n=5, *p* < 0.05 indicates that the experimental results are statistically significant).

Weight gain, adipose accumulation, and an increase in Lee’s index serve as intuitive gauges for assessing the success of obesity models and the efficacy of WP interventions. Furthermore, lipid profiles and liver histopathology act as vital indicators, reflecting the extent of lipid deposition within the organism. The liver and pancreas are the main organs of glucose and lipid metabolism in the organism and are also the main places where adipose tissue participates in energy metabolism. When the body’s energy intake is excessive and metabolism is not timely, it will lead to the deposition of lipids, causing an increase in the mass of the liver and pancreas and a large accumulation of adipose. As shown in **Figures 3A–D**, after 10 weeks of HFD intervention, compared with the control group, mice in the model group gained body weight, Lee’s index increased, liver coefficient, pancreas coefficient increased, and the weight of epididymal white adipose tissue (eWAT) was significantly elevated, after feeding high-fat chow and giving WP intervention at the same time, compared with the model group, the rate of body weight gain of mice in the WP administration group all slowed down, Lee’s index decreased, liver coefficient and pancreas coefficient decreased, and weight of epididymal eWAT decreased. As shown in **Figure 3E**, compared with the control group, the serum levels of TC, TG, and LDL-C in the model group were significantly increased, and the level of HDL-C was significantly decreased, after the WP intervention, the serum levels of TC, TG, and LDL-C in the mice were significantly decreased, and the level of HDL-C was significantly increased, and it was the most significant in the WP high-dose group. As shown in **Figure 3F**, the fasting blood glucose level of mice after consuming HFD increased significantly compared with the control group, and the fasting blood glucose level decreased after the administration of different doses of WP intervention, and the effect was the best in the high-dose group, but there was no dose-dependent trend. Meanwhile, to clarify whether WP affected the activity level and food intake of the mice, we performed Indirect Calorimetry Modular System (Oxylet, Propanlab) measurements. As shown in **Supplementary Figure 1**, the activity level, and the number of times of standing were increased in mice given WP intervention compared with the control and model groups, and the amount of food intake was slightly reduced compared with the control and model groups. control and model groups, a slight decrease in dietary intake, and an increase in water intake compared to the model group, which tended to be higher than that of the control group.

### 3.4 WP attenuates liver lesions and reduces adipocyte volume in HFD-fed mice

**Figure 4:**
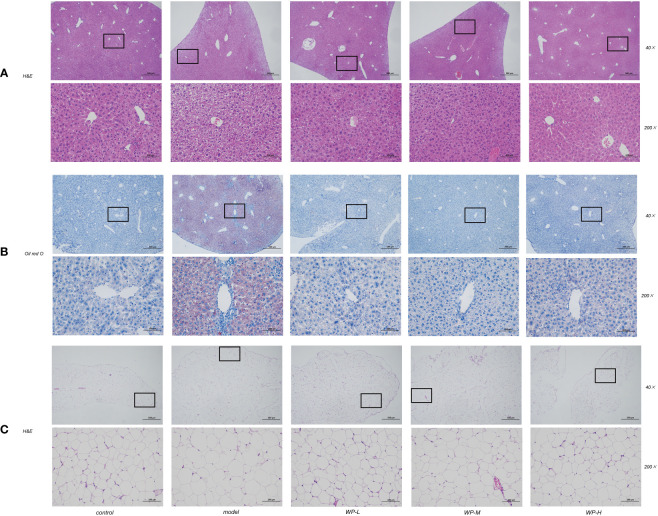
WP improves pathological changes of liver and adipose tissue in HFD-fed mice. **(A)** H&E staining of mice liver tissue to observe the effect of WP on the degree of vacuole-like changes and inflammatory cell infiltration in mice liver tissue (magnification 40 ×, 200 ×), **(B)** Oil red O staining of mice liver tissue to observe the effect of WP on lipid deposition in mice liver tissue (magnification 40 ×, 200 ×), **(C)** WAT of mice epididymis H&E staining to observe the effect of WP on adipocyte volume in mice (magnification 40 ×, 200 ×).

As shown in **Figures 4A, B**, liver pathology sections were stained with H&E staining and Oil Red O staining to assess histological liver injury. The liver tissue of the control group was morphologically and structurally normal, with no inflammatory cell infiltration as well as vacuole-like changes. Significant lobular inflammatory cell infiltration, vacuole-like changes, and lipid droplet deposition were observed in the liver tissues of mice in the model group compared to the control group. In contrast, the WP intervention significantly reduced these hepatic histological changes induced by HFD feeding and reduced hepatic vacuole changes and lipid droplet accumulation. We also found that the size of adipocytes in the eWAT of the epididymis of HFD-fed mice was significantly enlarged and disorganized compared with that of the control group and that the size of the adipocytes was reduced by WP treatment (**Figure 4C**).

### 3.5 WP reduces chronic metabolic inflammation levels and repairs the intestinal mucosal barrier in HFD-fed mice

A HFD can lead to an impaired intestinal mucosal barrier, increased levels of LPS into the bloodstream, stimulation of the proliferation of inflammatory factors in the organism through Toll-like Receptor 4 (TLR4), and impaired expression of sIgA, which in turn exacerbates the level of chronic metabolic inflammation in the organism.

**Figure 5:**
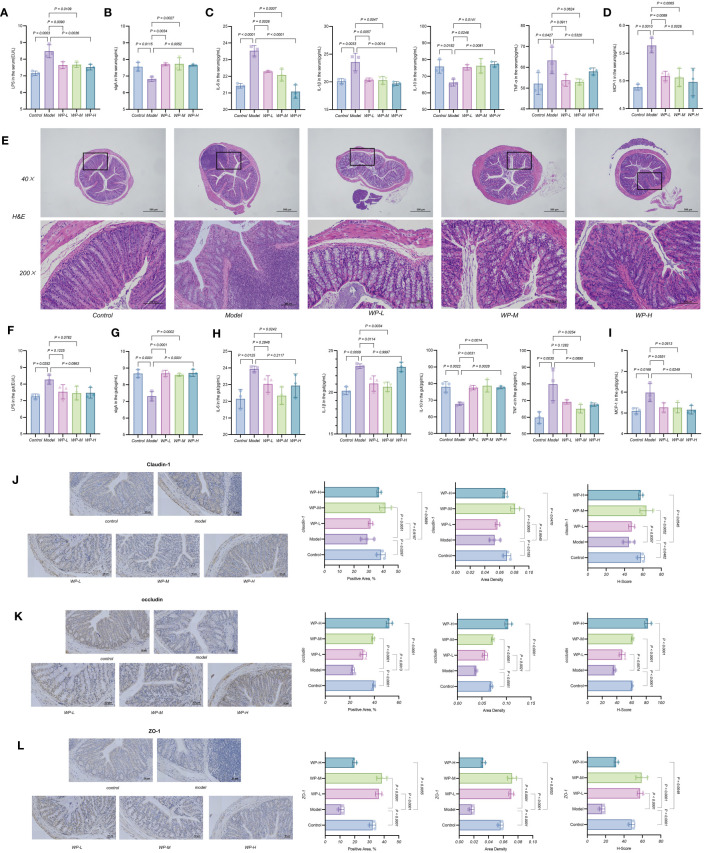
WP reduces chronic metabolic inflammation levels and repairs the intestinal mucosal barrier in HFD-fed mice. **(A-D)** ELISA to detect the effect of WP on the expression levels of LPS, sIgA, IL-6, IL-1β, IL-10, TNF-α and MCP-1 in mice serum, **(E)** H&E staining to observe the effect of WP on the degree of inflammatory infiltration and mucosal damage in colonic tissues (40×, 200×), **(F-I)** ELISA to detect the effect of WP on the expression levels of LPS, sIgA, IL-6, IL-1β, IL-10, TNF-α, and MCP-1 expression levels, **(J-L)** IHC to observe the effect of WP on the protein expression levels of Claudin-1, occludin, and ZO-1 in mice colon tissues and Positive Area, Area Density, and H-Score analysis. (n=3, *p* < 0.05 indicates that the experimental results are statistically significant).

As shown in **Figures 5A, B**, serum LPS content increased and sIgA expression decreased in the HFD model group of mice compared with the control group. However, after WP intervention, serum LPS content decreased and sIgA expression increased in mice. As shown in **Figures 5C, D**, compared with the model group, WP intervention significantly reduced the high expression of IL-6, IL-1β, TNF-α, and MCP-1 in the serum of HFD-fed mice, while elevating the expression of the inflammation-suppressing factor IL-10. The above results indicated that WP could effectively reduce the chronic metabolic inflammation level in HFD-fed mice organisms.

As shown in **Figure 5E**, H&E staining of colon tissues of control mice showed normal morphology and structure of intestinal tissues without significant inflammatory cell infiltration, whereas significant inflammatory cell infiltration was visible in HFD-fed mice intestinal tissues, but the above manifestations were ameliorated after WP intervention. The ELISA results of colon tissues were consistent with this, as shown in **Figures 5F–I**. HFD-fed mice colon tissues showed increased expression of LPS, decreased expression of sIgA, overexpression of inflammatory factors IL-6, IL-1β, TNF-α, and MCP-1, and decreased expression of inflammation-suppressing factor IL-10, after WP intervention, MCP-1 expression decreased, and IL-10 expression increased.

Claudin-1, occludin and ZO-1 proteins are important proteins for maintaining the integrity of the mechanical barrier of the intestinal mucosa. Immunohistochemistry of paraffin sections results in blue color staining of cytosolic hematoxylin and brownish-yellow color for DAB positive expression. As shown in **Figures 5J–L**, the expression levels of Claudin-1, occludin, and ZO-1 proteins were relatively down-regulated in the colonic tissues of mice in the model group compared with those in the control group, i.e., intestinal permeability was increased, and the integrity of the intestinal mucosal barrier was damaged. After the WP intervention, the expression levels of the tight junction proteins Claudin-1, occludin, and ZO-1 were increased. The expression levels of Claudin-1, occludin, and ZO-1 in the colonic tissues of mice increased and tended to be higher than those of the control group in terms of Positive Area, Area Density, and H-Score. These results suggest that WP can effectively reduce the inflammation level of colonic tissue in HFD-fed mice, increase the expression of tight junction proteins in colonic tissue, and then reduce the permeability of the intestinal mucosa and maintain the integrity of the intestinal mucosal barrier.

### 3.6 WP can modulate the relative abundance, diversity and structure of intestinal flora in HFD-fed mice

**Figure 6:**
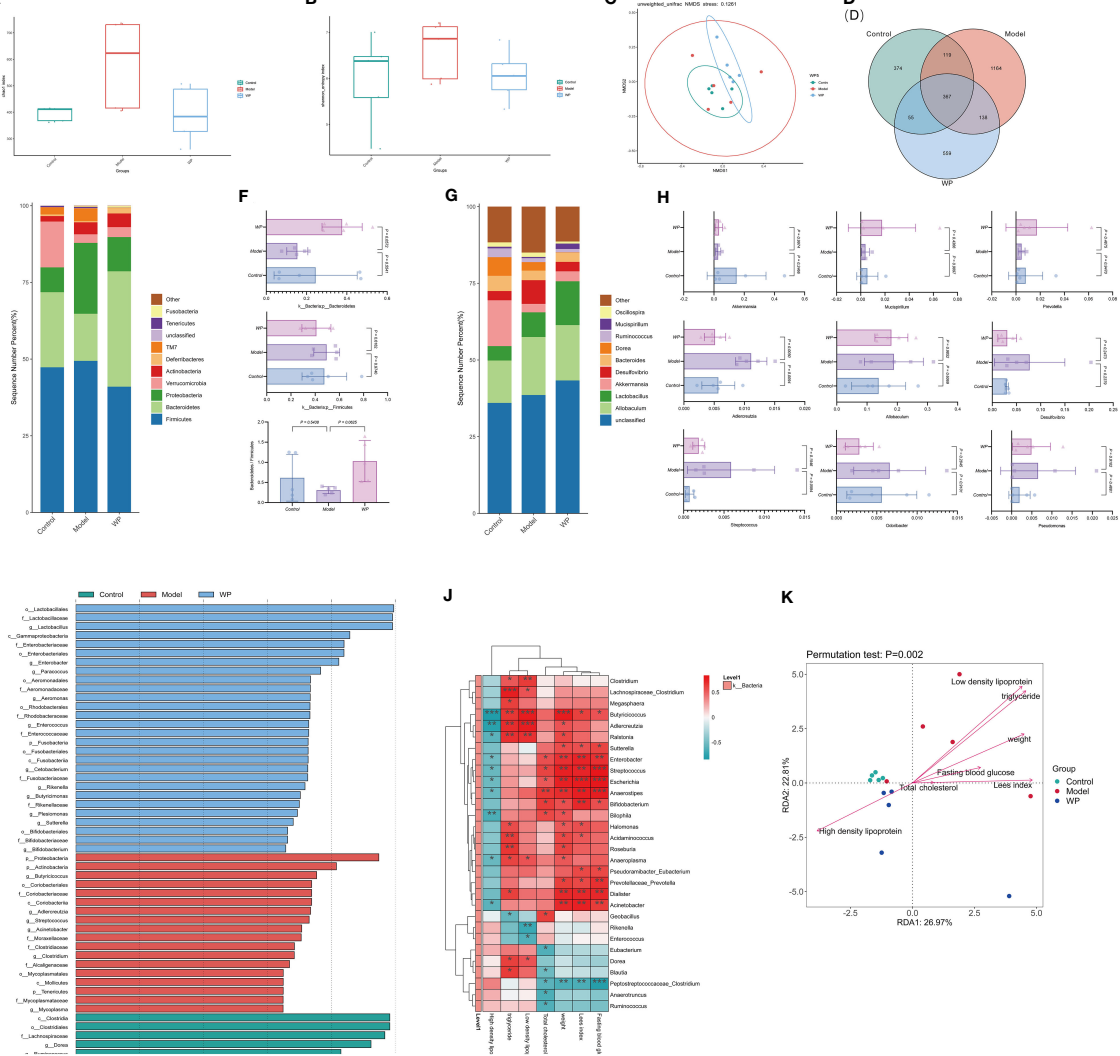
WP can modulate the relative abundance, diversity, and structure of intestinal flora in HFD-fed mice. **(A)** Chao 1 index to detect the effect of WP on the abundance of intestinal flora in mice, **(B)** Shannon index to detect the effect of WP on the diversity of intestinal flora in mice, **(C)** NMDS analysis to detect the effect of WP on the composition of intestinal flora in mice, **(D)** Venn diagram to demonstrate the number of intersecting flora and number of differing flora of enterobacteria in intestinal tracts of mice in each group, **(E)** the effect of WP at the phylum level on intestinal flora of mice effect, **(F)** Effect of WP at the phylumlevel on the Bacteroides, Firmicutes and Bacteroides/Firmicutes ratio, **(G)** Effect of WP at the genus level on the intestinal flora of mice, **(H)** Regulation of some intestinal phyla of mice by WP, **(I)** Effect of WP on the LDA value of the intestinal flora of mice, **(J)** Heatmap analyzing the relationship between the species of the intestinal flora at the genus level and the phenotype of related diseases (* 0.01 ≤ *P* < 0.05, ** 0.001 ≤ *P* < 0.01, *** *P* < 0.001), **(K)** Genus-level species RDA ordination plots analyzing the relationship between intestinal flora and environmental factors in mice. (n=5).

The Chao1 index and Shannon index can reflect the changes in the relative abundance and diversity of the flora. NMDS analyzed the differences between the samples of each group, and the farther the distance the greater the differences. As shown in **Figures 6A, B**, the relative abundance and diversity of the mice flora in the model group showed an increasing trend compared with the control group, and the relative abundance and diversity of the mice flora decreased significantly compared with the model group after WP intervention. It indicated that WP could inhibit the increase of relative abundance and diversity of HFD-fed mice intestinal flora. As shown in **Figure 6C**, by NMDS analysis, the samples of each group were spatially dispersed and arranged, suggesting that there was a large variability in the composition of the bacterial flora among the groups, in which the WP-intervention group was close to the control group, indicating that the structure of the intestinal bacterial flora of the mice tended to be in favor of that of the control group after the WP intervention. Meanwhile, as shown in **Figure 6D**, the Venn diagram was used to construct the intersection of the mice intestinal bacteria in each group and the differential flora was screened out at the same time.

As shown in **Figures 6E, F**, at the phylum level, the flora composition of the control group was dominated by the Bacteroides with a large Bacteroides/Firmicutes ratio, and the model group had an increased proportion of Firmicutes and Proteobacteria with a small Bacteroides/Firmicutes ratio, while the WP intervention increased the level of Bacteroides and decreased the level of Firmicutes, which resulted in a rise in the Bacteroides/Firmicutes ratio, and the change of the flora at the phylum level tended to be in favor of that in the control group. As shown in **Figures 6G, H**, analyzing the changes in the total abundance of the top-ranked dominant intestinal flora at the genus level in each group of mice by grouped percentage stacked bar charts, it is known that compared with the control group, the abundance of the genera of *Akkermansia*, *Mucispirillum*, and *Prevotella* showed a decreasing trend in the model group and that the abundance of the genera of *Adlercreutzia* was significantly increased, abundance tended to change towards the control group. As shown in **Supplementary Figure 2**, the top 50 genera with the highest abundance were selected to draw an evolutionary relationship tree diagram for species-specific phylogenetic analyses, with larger abundance-normalized values representing higher relative abundance. Larger LDA values in the LEfSe analyses indicated that the genus was more influential in the group. As shown in **Figure 6I**, the genera *Dorea*, *Ruminococcus*, *Clostridium*, *Blautia*, *Ruminococcus*, and *Eubacterium* were more influential in the control group, and *Butyricicoccus*, *Adlercreutzia*, and *Streptococcus*, *Acinetobacter* spp. was more influential in the model group, *Lactobacillus*, *Enterobacter*, *Paracoccus*, *Aeromonas*, *Enterococcus*, *Cetobacterium*, *Rikenella*, and *Butyricimonas* spp. in the WP group were more influential (LDA value > 3.5).

As shown in **Figure 6J**, the correlation analysis of glycemic and lipid metabolism-related disease phenotypes with intestinal flora species in each group of mice can reflect the interrelationships between intestinal microbial species and each metabolic phenotype at the genus level, and this result can screen out species that are significantly correlated with fasting glucose, Lee’s index, body weight, and lipid profiles. For example, *Butyricoccus bacteria* were significantly positively correlated with body weight, LDL, and triglycerides and significantly negatively correlated with HDL in mice. While *Streptococcus*, *Escherichia*, and *Anaerostipes bacteria* were significantly positively correlated with fasting glucose. *Peptostreptococcaceae_Clostridium bacteria* were negatively correlated with fasting glucose, Lee’s index, body weight, and total cholesterol. As shown in **Figure 6K**, in the genus-level species RDA ordination diagram, mice glycolipid metabolism-related disease phenotypes are indicated by arrows, and the longer the arrow, the greater the correlation between the phenotype and the distribution of communities and species. The angle between different phenotypes indicated the relationship between them, with an acute angle indicating a positive correlation and an obtuse angle indicating a negative correlation (*P* = 0.002), indicating that the glycolipid metabolism-related phenotypes of mice in each group had a significant effect on the intestinal microbial community. Meanwhile, the distance between the sample points of the model group and the control and WP groups was farther from the projection points in the graph, indicating a greater degree of difference in the structure of the flora.

### 3.7 WP modulates the SCFAs content of HFD-fed mice

**Figure 7:**
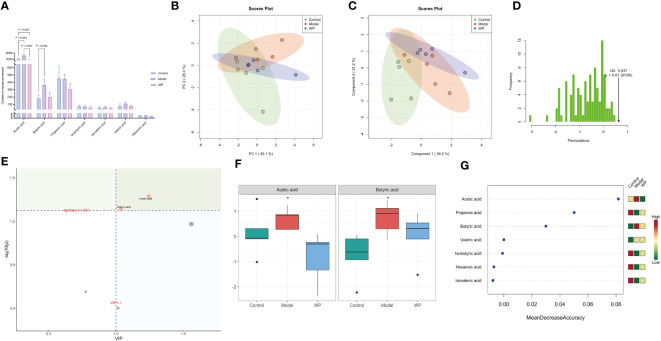
WP regulates the content of SCFAs in HFD-fed mice. **(A)** Histogram of the 7 SCFAs with the highest concentrations, **(B)** PCA plot of SCFAs in each group, **(C)** PLS-DA point cloud of SCFAs in each group, **(D)** Distribution of the test statistic (Q2) of the OPLS-DA replacement test and the P-value, **(E)** Importance plot of the metabolites of PLS-DA, **(F)** Effect of WP on the Acetic acid and Butyric acid content regulation (*, *P<*0.05), **(G)** The seven most important metabolites in random forests. (n=5).

Intestinal flora can regulate the immune and metabolic functions of the host through their metabolites, such as SCFAs. As shown in **Figure 7A**, there were differences in the content of SCFAs between the groups. This result was also supported by **Figures 7B–D**. The PCA and PLS-DA showed that the point cloud of each group was distributed in different regions, and the test statistic (Q2) of the OPLS-DA replacement test was on the right side of the random distribution with *P* < 0.01, suggesting that there were large differences in the compositional structure of SCFAs and the existence of metabolites with significant differences among the groups. As shown in **Figures 7E–G**, Acetic acid and Butyric acid were metabolites that differed significantly between subgroups at a corrected *P* < 0.05 and Value Importance in Projection (VIP) greater than one. Acetic acid and Butyric acid increased in the model group compared to the control group, and the levels tended to converge to the control group after the WP intervention. In addition, the results of Random Forest analysis showed that seven SCFAs differed between subgroups, and after representing the differences in the levels of SCFAs in each group by heat map, it was clear that, except for Acetic acid and Butyric acid, an HFD resulted in Propionic acid, Isobutyric acid, and Hexanoic acid, sovaleric acid, while the content of these four SCFAs tended to increase compared to the model group and converged to the level of the control group after the WP intervention.

## 4 Discussion

Numerous studies have substantiated that the moderate inclusion of walnuts in a diet does not result in weight gain (13), and can even decrease serum TG levels in patients with metabolic syndrome (14). Drawing on this evidence, we ventured to investigate the specific mechanisms of WP’s impact on reducing weight gain and modifying glycolipid metabolism triggered by a high-fat diet, leveraging animal studies. Simultaneously, we employed network pharmacology’s predictive results to discern other potential mechanisms and targets of WP. This is due to WP, an extract mixture from homologous walnuts, having a multi-target and multi-pathway effect in treating obesity and other conditions. This aligns with our experimental findings that WP can effectively decelerate weight gain and abnormal glucose-lipid metabolism in HFD-fed mice. The principal mechanism underlying these effects relates predominantly to the suppression of body inflammation and the remodeling of the intestinal microenvironment.

Chronic low-grade inflammation is an important feature of obesity, hypertrophy and expansion of adipocytes in the obese population, can cause local hypoxia, so that macrophages in the adipose tissue are enriched and further recruitment of more inflammatory factors (15), spreading obesity-related inflammatory state, and persistence, which in turn reduces the body’s immunity and induces metabolic-related diseases (16). Hypertrophy and expansion of adipocytes in the obese population can cause local hypoxia, which enriches macrophages in the adipose tissue and further recruits more inflammatory factors, propagating an obesity-associated inflammatory state that persists, which then reduces the body’s immune system and induces metabolism-related diseases. A large body of evidence suggests that inflammatory cytokines such as TNF-α, IL-1β, and IL-6 not only induce systemic insulin resistance but also affect lipid metabolism (17), whereas the presence of IL-10 effectively improves insulin sensitivity and HFD-induced obesity (18). Studies have shown that walnut-derived peptides can effectively prevent oxidative stress and inflammation of LPS-stimulated BV-2 microglia (11). In this study, WP attenuated the degree of hepatic inflammatory and fat vacuolization alterations in HFD-fed mice, inhibited the increase of adipose tissue and the enlargement of adipocytes in mice epididymis, and at the same time, it could be observed that the levels of LPS, IL-6, IL-1β, TNF-α, and MCP-1 expression in serum of obesity model mice were significantly decreased, and the expression of IL-10 was increased, while the level of the immune factor sIgA increased. Accordingly, regulating the expression of inflammatory factors and reducing the level of chronic low-grade inflammation in the organism is one of the mechanisms of WP to alleviate obesity. At the same time, we obtained 210 common potential targets including 50 core targets such as GAPDH, VEGFA, JUN, STAT3, SRC, etc. after taking the intersection of AMWP action targets with disease targets of obesity and inflammation, suggesting that WP can intervene in the development of obesity by acting on multiple inflammatory targets. This is consistent with previous research reports that WP can be anti-inflammatory.

In this study, we found that WP can repair the damaged intestinal mucosal barrier by regulating the expression level of inflammatory factors in the colon. The peptide leucine-proline-phenylalanine (LPF) has been found to alleviate colitis symptoms by reducing apoptosis, anti-inflammatory effects, and regulating gut microbiota (19), which is consistent with the findings of this study. Protein peptides extracted from walnuts can repair damaged intestinal mucosal barriers in mice with different disease models. The change in the intestinal microenvironment is one of the fastest and most intuitive responses of the body to changes in dietary structure. HFD can lead to many colonic inflammatory cell infiltrations, decreased colonic tight junction protein expression, and damage to the integrity of the intestinal mucosal barrier. LPS translocated into the bloodstream and binds to lipoproteins such as celiac disease, HDL, LDL, VLDL, etc. The resulting complexes are perfectively transported to the liver, where they are taken up by Kupffer cells, increasing TNF-alpha pre-production and further exacerbating inflammation. This in turn leads to suppression of liver metabolism and disruption of the body’s glucose and lipid levels (17). Thus, the low-grade inflammation present in obese individuals interacts with the intestinal microenvironment. The function of WP to regulate the intestinal microenvironment effectively reduces the level of inflammation in the body, and thus interferes with obesity and glucose and lipid metabolism.

The intestinal microbiota is a key regulator in the maintenance of homeostasis in its microenvironment, and imbalances in microbiota homeostasis can regulate host metabolism in multiple pathways to affect body weight by causing various pathological changes in the intestinal (15). Among them, the relative abundance ratios of the Firmicutes and Bacteroides are strongly associated with obesity (20), and one of the characteristics is the low Bacteroides/Firmicutes ratio (21, 22). The results of the present study were consistent with the report that the WP intervention increased the level of Bacteroides and decreased the level of Firmicutes in the intestinal flora of obese mice, and the ratio of Bacteroides/Firmicutes was up regulated. At the same time, the abundance of *Adlercreutzia* spp. positively correlated with BMI and inflammation was significantly reduced (23), and the genera of *Lactobacillus* and *Butyricimonas*, which have been demonstrated to be used for the prevention of obesity and the regulation of intestinal microecological balance, and which are capable of preventing HFD-fed mice diabetes and metabolic disorders via GLP-1 receptors, had a significant were all significantly increased in the overall bacterial genus group (17, 24, 25). Accordingly, in the mechanism of WP repairing the intestinal mucosal barrier to ameliorate obesity, in addition to the mechanical barrier, the regulation of the biological barrier also plays an important role.

In general, the higher the abundance and diversity of the intestinal flora, the better the health. In the case of obesity and other metabolic disorders, increased abundance and diversity may reflect an overgrowth of harmful bacteria and a decrease in beneficial bacteria. In our study, the increase in relative abundance and diversity of intestinal microbiota in the model group may indicate an ecological dysregulation state, possibly due to the shift of intestinal microbiota to a probiotic dysregulation state caused by a HFD (26), similar results have been found in human studies (27). On the other hand, WP treatment may restore a healthy balance by reducing the abundance and diversity of the intestinal microbiota by inhibiting the growth of these harmful bacteria and promoting the growth of beneficial bacteria. In this experiment, the abundance and diversity of intestinal microbiota in the WP group also tended to be higher than that in the control group. At the same time, studies have also confirmed that the nutrient intake of mice fed HFD is different from that of the control group, resulting in increased bacterial abundance in the HFD group (28). The intake of HFDs from different raw materials may lead to differences in intestinal flora diversity, and the interfering factors need to be further clarified in future experiments.

In this experiment, both Acetic acid and Butyric acid increased in the model group but decreased after WP intervention. Because fermentable fiber is in short supply in the gut, bacteria can also ferment proteins to produce certain substances, such as SCFAs, that can be harmful to the gut (29). However, at the same time, SCFAs level, as an important indicator to evaluate the intestinal microenvironment and the body’s energy metabolism level, has been shown to stimulate the production of leptin through related mechanisms, and inhibit appetite and energy intake through the gut-brain axis, thus intervening in the progression of obesity. Both Acetic acid and Butyric acid have been found to have higher levels in obese humans and rodents (30, 31). The content of Propionic acid (32), which has been proven to increase satiety, improve the body’s glucose homeostasis, inhibit inflammation, and improve obesity, in this experiment, after WP intervention, the trend was higher than that of the model group. Metatenomic studies of obesity have found that the pathway of SCFAs production is enriched, and the level of SCFAs is increased in overweight or obese human and animal models, which is consistent with these products of microbial fermentation providing extra calories to the host (33), in this experiment, we found that WP intervention reduces some SCFAs levels, it may be that blocking this pathway reduces the accumulation of energy. Many studies have also demonstrated that the microbiota metabolizes dietary fiber into SCFAs such as Butyric or Acetic, which enhance the integrity of the epithelial barrier and promote the resolution of intestinal inflammation (34), therefore, the effect of SCFAs produced by microorganisms on energy balance is also controversial (27). High levels of SCFAs increase energy absorption, but also have anti-inflammatory functions that repair the intestinal mucosal barrier, and further experimental investigation is needed to determine which function dominates in obese individuals.

Network pharmacology predicts that WP may regulate inflammation and ameliorate obesity through multiple pathways such as lipid and atherosclerosis, endocrine resistance, and diabetic cardiomyopathy. It means that WP has the potential to play a role in obesity, glycolipid metabolism, hepatopathy, intestinal inflammation, and intestinal microbiome. Active ingredients in WP, such as oxidized glutathione, may improve symptoms of HFD-induced obesity by acting on targets such as EGFR, NOS3, MMP2, PLG, PTGS2, and AR. Animal experiments have confirmed that WP may intervene in obesity by regulating the level of intestinal inflammation and intestinal microbes, etc. Whether WP is related to the effects of EGFR, NOS3, MMP2, PLG, PTGS2, AR and other targets needs to be further verified by future experiments. oxidized glutathione is a powerful antioxidant that can potentially counteract the negative effects of a HFD on the intestinal microbiome, and there have also been studies reported on the mechanism by which glutathione improves obesity primarily related to glycine-conjugated bile acid metabolism (35). Adding or removing components such as oxidized glutathione from WP group feeds may also provide more insight into the possible mechanisms of WP’s anti-obesity and anti-inflammatory effects. We will consider this in future experiments.

To sum up, obesity is directly associated with persistent inflammation and imbalances in the intestinal microenvironment. Plant-derived peptide WP can mitiphylumthese issues by restoring the integrity of the intestinal mucosal barrier and by modifying the intestinal ecosystem. This is achieved through the regulation of tight junction proteins in the intestine, correction of intestinal bacterial flora imbalance, and altering the content of SCFAs metabolites. Consequently, WP reduces inflammation, slows weight gain in HFD fed mice, and corrects abnormalities in glucose-lipid metabolism. It also alleviates pathological changes in various organs, including the liver, pancreas, and colon, potentially caused by related diseases. Oxidized glutathione might be the key active ingredient contributing to its effectiveness. Previous studies have reported that WP has higher nutritional, functional, and essential amino acids compared to other plant and grain proteins. The main activities are antihypertensive, antioxidant, learning improvement and anticancer (36). Our research provides empirical evidence that may broaden the use of WP as a dietary supplement for obesity management and regulation of glycolipid metabolic disorders. These findings endorse the use of WP as a prospective therapeutic agent for preventing and treating obesity and correlated metabolic disorders. However, further studies are required to unravel the precise mechanism through which WP has its anti-obesity effects. Additionally, clinical trials are necessary to validate the safety and efficacy of WP in human applications.

## Data availability statement

The datasets presented in this study can be found in online repositories. The names of the repository/repositories and accession number(s) can be found here: https://www.ncbi.nlm.nih.gov/sra, accession number PRJNA1022998.

## Ethics statement

The animal study was approved by Animal Ethical Experiments Committee of Yunnan University of Chinese Medicine (Approval number: R-062021084). The study was conducted in accordance with the local legislation and institutional requirements.

## Author contributions

LL: Conceptualization, Data curation, Formal analysis, Methodology, Project administration, Software, Writing – original draft, Writing – review & editing. SW: Data curation, Methodology, Writing – original draft. TZ: Formal analysis, Writing – review & editing. BL: Investigation, Writing – review & editing. YJ: Data curation, Methodology, Writing – review & editing. YWa: Formal analysis, Writing – review & editing. XC: Methodology, Writing – review & editing. NL: Data curation, Formal analysis, Writing – review & editing. NH: Project administration, Resources, Writing – review & editing. YWu: Data curation, Funding acquisition, Methodology, Resources, Writing – review & editing. JY: Formal analysis, Funding acquisition, Project administration, Visualization, Writing – review & editing.
