# Supplementary material for: Walnut peptide alleviates obesity, inflammation and dyslipidemia in mice fed a high-fat diet by modulating the intestinal flora and metabolites
Source: Front Immunol. 2023 Dec 14;14:1305656. doi: 10.3389/fimmu.2023.1305656 (PMC10755907; doi:10.3389/fimmu.2023.1305656)
Supplement: Supplementary file 1 [file DataSheet_1.docx]

**Supplementary material 1 Immunohistochemical antibody information and repair conditions**

| Antigen repair condition | Name | Article No. | Manufacturer | Species | Dilution ratio | Name of the corresponding secondary antibody |
| --- | --- | --- | --- | --- | --- | --- |
| EDTA antigen repair solution (pH 9.0) moderate fire 8min, ceasefire 8min, moderate low fire 7min | Claudin1 | GB11032 | Servicebio | Rabbit | 1:800 | HRP was used to label goat anti-rabbit secondary antibody |
| EDTA antigen repair solution (pH 9.0) moderate fire 8min, ceasefire 8min, moderate low fire 7min | ZO-1 | GB111402 | Servicebio | Rabbit | 1:1000 | HRP was used to label goat anti-rabbit secondary antibody |
| EDTA antigen repair solution (pH 9.0) moderate fire 8min, ceasefire 8min, moderate low fire 7min | Occludin | GB111401 | Servicebio | Rabbit | 1:1000 | HRP was used to label goat anti-rabbit secondary antibody |
