# Supplementary material for: Walnut peptide alleviates obesity, inflammation and dyslipidemia in mice fed a high-fat diet by modulating the intestinal flora and metabolites
Source: Front Immunol. 2023 Dec 14;14:1305656. doi: 10.3389/fimmu.2023.1305656 (PMC10755907; doi:10.3389/fimmu.2023.1305656)
Supplement: Supplementary file 2 [file DataSheet_2.docx]

**Supplementary material 2** **Second antibody article number, manufacturer, and dilution ratio**

| Second Antibody and TSA name | Article number | Manufacturer | Dilution ratio |
| --- | --- | --- | --- |
| Cy3 conjugated Goat Anti-Rabbit IgG | GB21303 | Servicebio | 1:300 |
| CY3 conjugated Goat Anti-Mouse IgG | GB21301 | Servicebio | 1:300 |
| CY3 conjugated Goat Anti-Rat IgG | GB21302 | Servicebio | 1:300 |
| CY3 conjugated Donkey Anti-Goat IgG | GB21404 | Servicebio | 1:300 |
| Cy3 conjugated Donkey Anti-Mouse IgG | GB21401 | Servicebio | 1:300 |
| CY3 conjugated Donkey Anti-Rabbit IgG | GB21403 | Servicebio | 1:300 |
| Alexa Fluor 488 conjugated Goat Anti-Rabbit IgG | GB25303 | Servicebio | 1:400 |
| Alexa Fluor 488 conjugated Goat Anti-Mouse IgG | GB25301 | Servicebio | 1:400 |
| CY5 conjugated Goat Anti-Mouse IgG | GB27301 | Servicebio | 1:400 |
| CY5 conjugated Goat Anti-Rabbit IgG | GB27303 | Servicebio | 1:400 |
| HRP conjugated Rabbit Anti-Goat IgG | GB23204 | Servicebio | 1:200 |
| HRP conjugated Goat Anti-Mouse IgG | GB23301 | Servicebio | 1:200 |
| HRP conjugated Goat Anti-Rat IgG | GB23302 | Servicebio | 1:200 |
| HRP conjugated Goat Anti-Rabbit IgG | GB23303 | Servicebio | 1:200 |
| HRP conjugated Donkey Anti-Goat IgG | GB23404 | Servicebio | 1:200 |
| FITC conjugated Donkey Anti-Goat IgG | GB22404 | Servicebio | 1:200 |
| FITC conjugated Goat Anti-Rat IgG | GB22302 | Servicebio | 1:200 |
| FITC conjugated Donkey Anti- Rabbit IgG | GB22403 | Servicebio | 1:200 |
| FITC conjugated Donkey Anti- Mouse IgG | GB22401 | Servicebio | 1:200 |
| Alexa Fluor 594 conjugated Goat Anti-Rabbit IgG | 111-585-003 | Jackson | 1:400 |
| Alexa Fluor 594 conjugated Goat Anti- Mouse IgG | 115-585-003 | Jackson | 1:400 |
| CY3-Tyramide | G1223 | Servicebio | 1:500 |
| iF488-Tyramide | G1231 | Servicebio | 1:500 |
| iF647-Tyramide | G1232 | Servicebio | 1:400 |
| FITC-Tyramide | G1222 | Servicebio | 1:500 |
