# Supplementary material for: Walnut peptide alleviates obesity, inflammation and dyslipidemia in mice fed a high-fat diet by modulating the intestinal flora and metabolites
Source: Front Immunol. 2023 Dec 14;14:1305656. doi: 10.3389/fimmu.2023.1305656 (PMC10755907; doi:10.3389/fimmu.2023.1305656)
Supplement: Supplementary file 3 [file DataSheet_3.docx]

**Supplementary material 3:Amino acids and their metabolites in WP for LC-MS/MS detection**

| Compounds | Class | Q1 (Da) | Q3 (Da) | Molecular Weight | Ion mode | Ionization model | Formula | sample-1 | sample-2 | sample-3 |
| --- | --- | --- | --- | --- | --- | --- | --- | --- | --- | --- |
| Phosphorylethanolamine | - | 142.026 | 44.049 | 141.06 | Positive | [M+H]+ | C2H8NO4P | 99032.4056 | 120443.238 | 110145.698 |
| L-Alanine | Amino Acid metabolomics | 90.05 | 44.05 | 89.05 | Positive | [M+H]+ | C3H7NO2 | 990280.318 | 1100207.31 | 1022937.59 |
| L-Aspartate | Amino Acid metabolomics | 134.04 | 74 | 133.10 | Positive | [M+H]+ | C4H7NO4 | 968634.195 | 1096150.05 | 1035223.47 |
| L-Asparagine Anhydrous | Amino Acid metabolomics | 133.06 | 74 | 132.05 | Positive | [M+H]+ | C4H8N2O3 | 959313.121 | 1033326.75 | 947781.059 |
| L-α-Aspartyl-L-phenylalanine | Amino Acid metabolomics | 281.3 | 166 | 280.11 | Positive | [M+H]+ | C13H16N2O5 | 956832.008 | 1093326.75 | 1005404.61 |
| Homo-L-arginine | Amino Acid metabolomics | 189 | 144 | 188.13 | Positive | [M+H]+ | C7H16N4O2 | 9203.90656 | 9598.73643 | 9045.40264 |
| D-Alanyl-D-Alanine | Amino Acid metabolomics | 161.09 | 44.04 | 160.09 | Positive | [M+H]+ | C6H12N2O3 | 917255.467 | 1041510.37 | 1004646.58 |
| L-Cystathionine | Amino Acid metabolomics | 223.07 | 134 | 222.07 | Positive | [M+H]+ | C7H14N2O4S | 9170.33797 | 10304.3435 | 9123.82359 |
| L-Histidine | Amino Acid metabolomics | 156.07 | 110.07 | 155.07 | Positive | [M+H]+ | C6H9N3O2 | 910336.978 | 1011421.52 | 960452.845 |
| 5-Hydroxy-Tryptamine | Amino Acid metabolomics | 177.1 | 160 | 176.21 | Positive | [M+H]+ | C10H12N20 | 8938.28032 | 10559.8223 | 11390.3327 |
| 4-Acetamidobutyric Acid | Organic Acid And Its Derivatives | 146.074 | 86 | 145.07 | Positive | [M+H]+ | C6H11NO3 | 8452.23658 | 10100 | 8856.78283 |
| S-(5-Adenosyl)-L-Homocysteine | Amino Acid metabolomics | 385 | 136.06 | 384.12 | Positive | [M+H]+ | C14H20N6O5S | 837.774354 | 731.068115 | 750.546367 |
| Trans-4-Hydroxy-L-Proline | Amino Acid metabolomics | 132.058 | 86 | 131.06 | Positive | [M+H]+ | C5H9NO3 | 7666.87873 | 7293.20829 | 6320.73243 |
| L-Proline | Amino Acid metabolomics | 116.1 | 70.1 | 115.06 | Positive | [M+H]+ | C5H9NO2 | 74307.1571 | 89423.9882 | 78444.0835 |
| L-Carnosine | Amino Acid metabolomics | 227.2 | 156 | 226.11 | Positive | [M+H]+ | C9H14N4O3 | 733697.813 | 823176.703 | 797061.429 |
| L-Cystine | Amino Acid metabolomics | 239 | 120 | 240.02 | Negative | [M-H]- | C6H12N2O4S2 | 71823.8569 | 73146.4956 | 69741.583 |
| Kinurenine | Organic Acid And Its Derivatives | 209.1 | 146 | 208.09 | Positive | [M+H]+ | C10H12N2O3 | 710.090457 | 881.035538 | 770.713723 |
| 5-Aminovaleric Acid | Organic Acid And Its Derivatives | 118.2 | 55 | 117.08 | Positive | [M+H]+ | C5H11NO2 | 70870.7753 | 81306.1204 | 75975.6842 |
| 3-Iodo-L-Tyrosine | Amino Acid metabolomics | 308.2 | 261.97 | 306.97 | Positive | [M+H]+ | C9H10INO3 | 705.710736 | 908.446199 | 795.445954 |
| Methionine Sulfoxide | Amino Acid metabolomics | 166 | 74 | 165.05 | Positive | [M+H]+ | C5H11NO3S | 68221.3718 | 74638.4995 | 69664.5009 |
| argininosuccinic acid | Amino Acid metabolomics | 291.5 | 176.1 | 290.27 | Positive | [M+H]+ | C10H18N4O6 | 6492296.22 | 7587384.01 | 7096190.19 |
| Glutathione Oxidized | Amino Acid metabolomics | 613.152 | 484 | 612.15 | Positive | [M+H]+ | C20H32N6O12S2 | 64788.668 | 63096.0513 | 60303.5046 |
| Kynurenic Acid | Organic Acid And Its Derivatives | 190.043 | 144 | 189.04 | Positive | [M+H]+ | C10H7NO3 | 63.2416501 | 76.6500494 | 79.7793857 |
| L-Serine | Amino Acid metabolomics | 106.04 | 60 | 105.04 | Positive | [M+H]+ | C3H7NO3 | 593183.897 | 660846.989 | 641195.117 |
| 3-Hydroxyhippuric Acid | Organic Acid And Its Derivatives | 196.05 | 121 | 195.05 | Positive | [M+H]+ | C9H9NO4 | 56.8904573 | 78.810464 | 59.1915731 |
| Succinic Acid | Amino Acid metabolomics | 117.03 | 99 | 118.03 | Negative | [M-H]- | C4H6O4 | 5571391.65 | 5075577.49 | 4672652.1 |
| Beta-Alanine | Amino Acid metabolomics | 90.2 | 30 | 89.05 | Positive | [M+H]+ | C3H7NO2 | 5189.79125 | 5945.88351 | 5247.64717 |
| Creatine Phosphate | Organic Acid And Its Derivatives | 212.04 | 114.06 | 211.11 | Positive | [M+H]+ | C4H8N3Na2O5P | 502964.215 | 605974.334 | 474509.746 |
| S-Sulfo-L-Cysteine | Amino Acid metabolomics | 201.98 | 120 | 200.98 | Positive | [M+H]+ | C3H7NO5S2 | 49723.161 | 55006.4166 | 48959.5393 |
| L-Methionine | Amino Acid metabolomics | 150.05 | 104 | 149.05 | Positive | [M+H]+ | C5H11NO2S | 484073.559 | 542103.653 | 501355.582 |
| L-Citrulline | Amino Acid metabolomics | 176.1 | 113 | 175.10 | Positive | [M+H]+ | C6H13N3O3 | 478888.668 | 547432.379 | 497382.359 |
| N-Isovaleroylglycine | Amino Acid metabolomics | 158.1 | 74 | 159.09 | Negative | [M-H]- | C7H13NO3 | 436.465209 | 403.416584 | 380.46269 |
| 5-Hydroxylysine | Amino Acid metabolomics | 163.1 | 128.07 | 162.18 | Positive | [M+H]+ | C6H14N2O3 | 4346.86879 | 4665.08391 | 4432.66391 |
| L-Homocitrulline | Amino Acid metabolomics | 190.1 | 127 | 189.11 | Positive | [M+H]+ | C7H15N3O3 | 4071.91849 | 4722.95163 | 4407.75743 |
| α-Aminoadipic acid | Organic Acid And Its Derivatives | 162.07 | 98.06 | 161.16 | Positive | [M+H]+ | C6H11NO4 | 4006.36183 | 3700.1382 | 3901.47667 |
| Trimethylamine N-Oxide | Amino Acid metabolomics | 76 | 58.1 | 75.07 | Positive | [M+H]+ | C3H9NO | 4.59514911 | 5.14892399 | 4.80494192 |
| Glycine | Amino Acid metabolomics | 76.03 | 30 | 75.03 | Positive | [M+H]+ | C2H5NO2 | 392343.936 | 412126.357 | 357075.212 |
| N-Glycyl-L-Leucine | Amino Acid metabolomics | 189.1 | 86 | 188.12 | Positive | [M+H]+ | C8H16N2O3 | 3912972.17 | 4381599.21 | 4163240.8 |
| 3-N-Methyl-L-Histidine | Amino Acid metabolomics | 170 | 96 | 169.18 | Positive | [M+H]+ | C7H11N3O2 | 3881.0338 | 4560.9773 | 4151.22071 |
| L-Tyrosine | Amino Acid metabolomics | 182.08 | 136.07 | 181.19 | Positive | [M+H]+ | C9H11NO3 | 3792117.3 | 4221776.9 | 3885843.67 |
| Sarcosine | Amino Acid metabolomics | 90.2 | 44.1 | 89.05 | Positive | [M+H]+ | C3H7NO2 | 3772.13718 | 4429.35834 | 3539.45659 |
| L-Arginine | Amino Acid metabolomics | 175.1 | 70.06 | 174.11 | Positive | [M+H]+ | C6H14N4O2 | 3629423.46 | 4126633.76 | 3840992.32 |
| L-Threonine | Amino Acid metabolomics | 120.06 | 74 | 119.06 | Positive | [M+H]+ | C4H9NO3 | 346151.093 | 381264.561 | 343262.453 |
| Urea | Amino Acid metabolomics | 61 | 44 | 60.03 | Positive | [M+H]+ | CH4N2O | 339102.386 | 383443.238 | 356235.479 |
| L-Tryptophyl-L-glutamic acid | Amino Acid metabolomics | 334 | 159 | 333.13 | Positive | [M+H]+ | C16H19N3O5 | 3335705.77 | 3391964.46 | 3379582.59 |
| 3,7-Dimethyluric Acid | Organic Acid And Its Derivatives | 195 | 180 | 196.06 | Negative | [M-H]- | C7H8N4O3 | 33.1595427 | 29.1992103 | 23.0534554 |
| L-Leucine | Amino Acid metabolomics | 132.1 | 86.1 | 131.10 | Positive | [M+H]+ | C6H13NO2 | 3253220.68 | 3605834.16 | 3325585.75 |
| Nα-Acetyl-L-Arginine | Amino Acid metabolomics | 217.1 | 158 | 216.12 | Positive | [M+H]+ | C8H16N4O3 | 32393.9364 | 34493.1885 | 32857.0585 |
| 6-Aminocaproic Acid | Organic Acid And Its Derivatives | 132.095 | 69 | 131.10 | Positive | [M+H]+ | C6H13NO2 | 31605.5666 | 29920.1382 | 28689.7027 |
| N-Acetylneuraminic Acid | Amino Acid metabolomics | 310.11 | 274.09 | 309.11 | Positive | [M+H]+ | C11H19NO9 | 29870.6759 | 29045.8045 | 28393.483 |
| Creatine | Organic Acid And Its Derivatives | 132.1 | 90 | 131.07 | Positive | [M+H]+ | C4H9N3O2 | 286.403579 | 308.849951 | 281.549518 |
| 3-Aminoisobutanoic Acid | Amino Acid metabolomics | 104.07 | 86.05 | 103.06 | Positive | [M+H]+ | C4H9NO2 | 2659.08549 | 2538.71668 | 2847.50935 |
| L-Phenylalanine | Amino Acid metabolomics | 166.1 | 120.08 | 165.08 | Positive | [M+H]+ | C9H11NO2 | 2474343.94 | 2771352.42 | 2584475.29 |
| glycylphenylalanine | Amino Acid metabolomics | 223.2 | 120.2 | 222.10 | Positive | [M+H]+ | C11H14N2O3 | 244293.241 | 278334.65 | 256948.218 |
| 2-Aminoethanesulfonic Acid | Organic Acid And Its Derivatives | 124 | 80 | 125.15 | Negative | [M-H]- | C2H7NO3S | 238.166004 | 235.529121 | 221.204962 |
| (5-L-Glutamyl)-L-Amino Acid | Amino Acid metabolomics | 219.09 | 202 | 218.09 | Positive | [M+H]+ | C8H14N2O5 | 223023.857 | 252961.5 | 234282.339 |
| Glycyl-L-Proline | Amino Acid metabolomics | 173.1 | 116.071 | 172.09 | Positive | [M+H]+ | C7H12N2O3 | 2221.68986 | 2332.53702 | 2257.24552 |
| L-Tryptophan | Amino Acid metabolomics | 205.1 | 118.06 | 204.09 | Positive | [M+H]+ | C11H12N2O2 | 21742842.9 | 23736722.6 | 22497243.6 |
| L-Lysine | Amino Acid metabolomics | 147.069 | 84 | 146.11 | Positive | [M+H]+ | C6H14N2O2 | 2114532.8 | 2294136.23 | 2201063.2 |
| 2-Aminobutyric acid | Organic Acid And Its Derivatives | 104.1 | 58.14 | 103.12 | Positive | [M+H]+ | C4H9NO2 | 178769.384 | 193667.325 | 178114.786 |
| L-Isoleucine | Amino Acid metabolomics | 132.1 | 86.1 | 131.10 | Positive | [M+H]+ | C6H13NO2 | 1585337.97 | 1738065.15 | 1616794.64 |
| L-Valine | Amino Acid metabolomics | 118.1 | 72.1 | 117.08 | Positive | [M+H]+ | C5H11NO2 | 1526789.26 | 1690138.2 | 1561399.88 |
| O-Phospho-L-Serine | Amino Acid metabolomics | 186 | 88.2 | 185.01 | Positive | [M+H]+ | C3H8NO6P | 14577.9324 | 19120.7305 | 16130.1437 |
| N-Acetyl-L-Tyrosine | Amino Acid metabolomics | 224 | 136 | 223.09 | Positive | [M+H]+ | C11H13NO4 | 14522.664 | 17397.1372 | 16666.8636 |
| Ethanolamine | - | 62 | 44 | 61.08 | Positive | [M+H]+ | C2H7NO | 13802.8827 | 15365.2517 | 13585.6468 |
| L-Glutamic acid | Amino Acid metabolomics | 148.06 | 84 | 147.05 | Positive | [M+H]+ | C5H9NO4 | 12485188.9 | 14283218.2 | 12970368.2 |
| N-Propionylglycine | Amino Acid metabolomics | 130.1 | 74 | 131.06 | Negative | [M-H]- | C5H9NO3 | 1217.65408 | 1084.36328 | 927.490648 |
| N-Acetylaspartate | Amino Acid metabolomics | 176 | 134 | 175.05 | Positive | [M+H]+ | C6H9NO5 | 120846.918 | 133879.566 | 162834.219 |
| γ-Glutamate-Cysteine | Amino Acid metabolomics | 251.07 | 130.05 | 250.06 | Positive | [M+H]+ | C8H14N2O5S | 12033.7972 | 8244.47187 | 11368.478 |
| 1-Methylhistidine | Amino Acid metabolomics | 170.1 | 124 | 169.09 | Positive | [M+H]+ | C7H11N3O2 | 11876.839 | 12807.6012 | 12105.9264 |
| Nα-Acetyl-L-glutamine | Amino Acid metabolomics | 189.1 | 130.1 | 188.18 | Positive | [M+H]+ | C7H12N2O4 | 11582.6044 | 13673.4452 | 12555.2274 |
| N6-Acetyl-L-Lysine | Amino Acid metabolomics | 189.1 | 126 | 188.12 | Positive | [M+H]+ | C8H16N2O3 | 1095.81511 | 1033.39585 | 1188.64934 |
| L-tyrosine methyl ester | Amino Acid metabolomics | 196.2 | 136.3 | 195.09 | Positive | [M+H]+ | C10H13NO3 | 1081.5507 | 1249.80257 | 1144.58555 |
| L-Ornithine | Amino Acid metabolomics | 133.09 | 70 | 132.16 | Positive | [M+H]+ | C5H13ClN2O2 | 1025516.9 | 1121342.55 | 1093965.35 |
| 5-Hydroxy-tryptophan | Amino Acid metabolomics | 219.1 | 74 | 220.22 | Negative | [M-H]- | C11H12N2O3 | 10160.5368 | 12134.7483 | 12128.1748 |
| L-Glutamine | Amino Acid metabolomics | 147.069 | 84 | 146.07 | Positive | [M+H]+ | C5H10N2O3 | 1012713.72 | 1091105.63 | 1031689.31 |
| γ-Aminobutyric Acid | Organic Acid And Its Derivatives | 104.06 | 68.8 | 103.06 | Positive | [M+H]+ | C4H9NO2 | 10015.6064 | 14598.618 | 13891.5141 |
