# Supplementary figures and images for: Walnut peptide alleviates obesity, inflammation and dyslipidemia in mice fed a high-fat diet by modulating the intestinal flora and metabolites
Source: Front Immunol. 2023 Dec 14;14:1305656. doi: 10.3389/fimmu.2023.1305656 (PMC10755907; doi:10.3389/fimmu.2023.1305656)

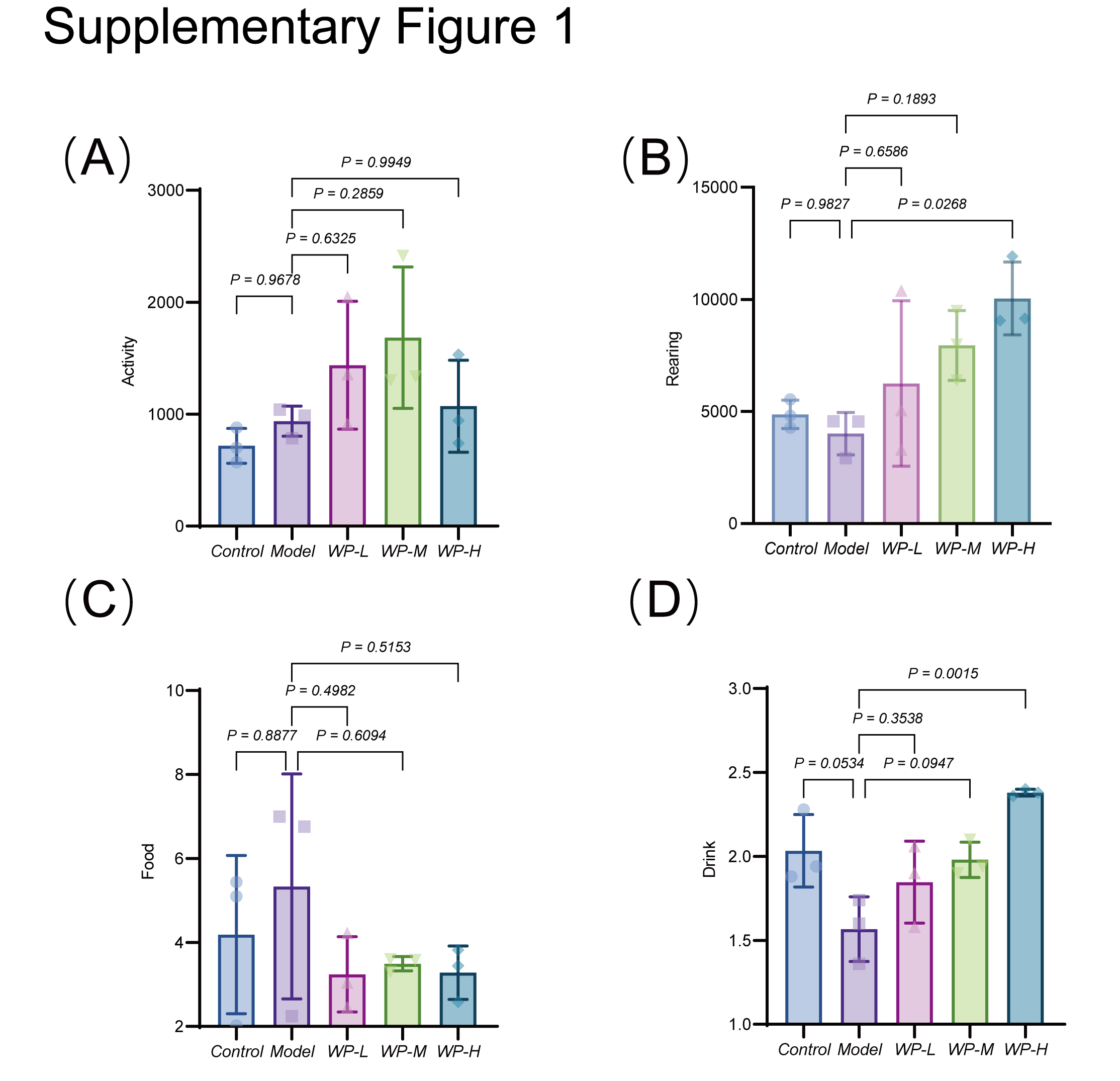

Supplement: Supplementary file 4 [file Image_1.jpg]
