# Supplementary material for: Walnut peptide alleviates obesity, inflammation and dyslipidemia in mice fed a high-fat diet by modulating the intestinal flora and metabolites
Source: Front Immunol. 2023 Dec 14;14:1305656. doi: 10.3389/fimmu.2023.1305656 (PMC10755907; doi:10.3389/fimmu.2023.1305656)
Supplement: Supplementary file 5 [file Image_2.pdf]

Supplementary Material

Supplementary Figure 2

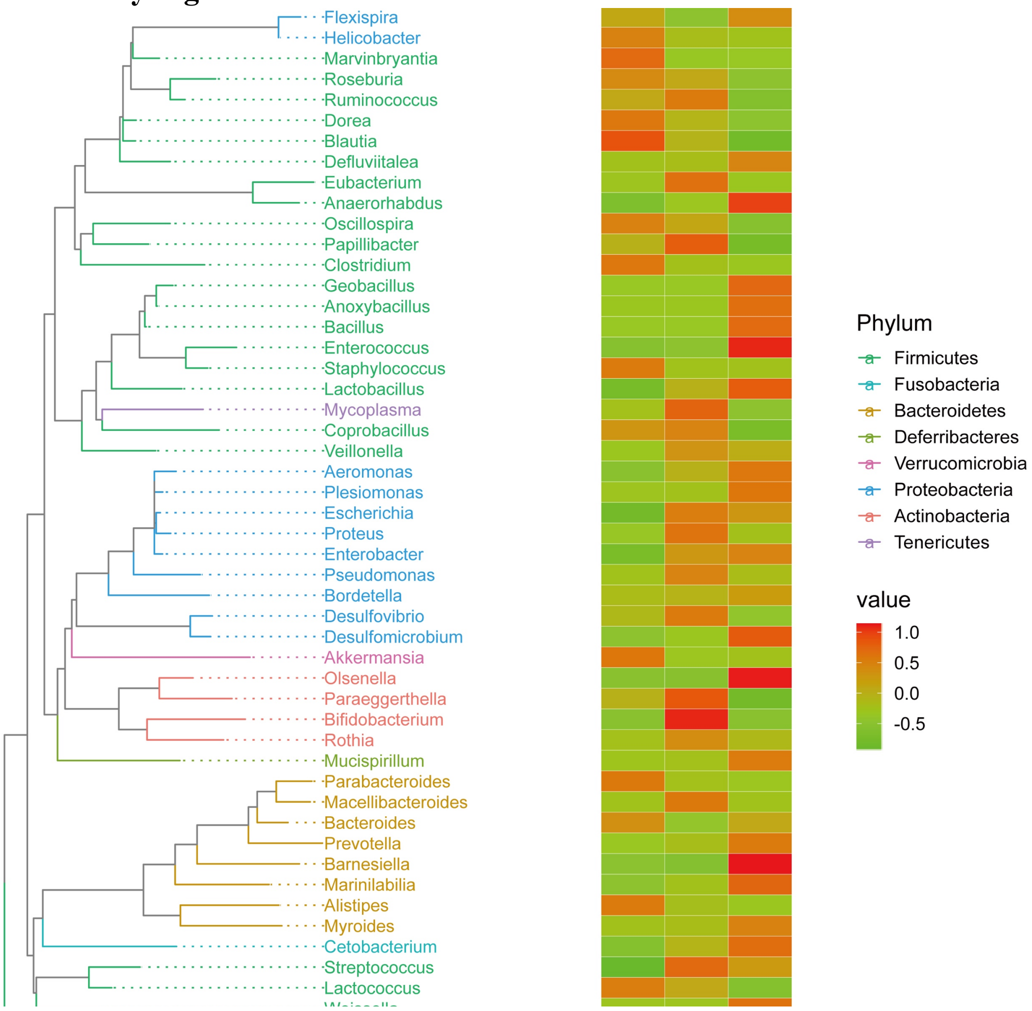

Supplementary Figure 2. Evolutionary relationship tree mapping of the top 50 most abundant genera of bacteria.

The top 50 genera with the highest abundance were selected to draw evolutionary relationship tree for interspecific phylogenetic analysis. The larger the normalized abundance value, the higher the relative abundance.
